# Clinical Profile and Outcome in Children with Post Diphtheritic Paralysis in a Tertiary Care Hospital in Southern India

**DOI:** 10.22037/ijcn.v16i1.23092

**Published:** 2022-03-14

**Authors:** Vykuntaraju K Gowda, Sukanya Vignesh, Asha Benakappa, Naveen Benakappa, Sanjay K Shivappa

**Affiliations:** 1 Department of Pediatric Neurology, Indira Gandhi Institute of Child Health, Bangalore , Indira; 2 Department of Pediatrics, Indira Gandhi Institute of Child Health, Bangalore, Indira

**Keywords:** Post-Diphtheritic Paralysis (PDP), anti-diphtheritic serum, diphtheria

## Abstract

**Objectives:**

Post-Diphtheritic Paralysis (PDP), one of the most severe complications of diphtheria, is caused by exotoxin of Corynebacterium diphtheria. This study was planned since there has been a resurgence of diphtheria in India in recent years due to a number of epidemiological factors.

**Materials & Methods:**

Thirty-five children with PDP were studied in a tertiary care hospital in Southern India**. **

**Result:**

Neurological complications occurred in 38.5% of 91 patients with faucial diphtheria. Of the patients, 13 (37.1%) were unimmunized, 12 (34.3%) were partially immunized, two (5.7%) were completely immunized, and eight (22.6%) had unknown status. Isolated bulbar palsy and bulbar palsy followed by limb weakness were seen in 20 (57.1%) and 15 (42.9%) of the patients, respectively. The first symptoms of PDP occurred 5-34 days after the onset of local diphtheria infection. Eleven (31.4%) out of the 35 patients had received antitoxin between days 5-7 of illness. Ventilation-dependent respiratory failure occurred in three (8.6%) patients with PDP. Nine (25.7%) patients had evidence of co-existent myocarditis, while myocarditis with renal failure was seen in two (5.7%) patients. Four (11.4%) patients died, three from severe cardiomyopathy and one from aspiration. Demyelinating neuropathy was noted in 64% of the patients. Children with bulbar palsy recovered in 4-7 weeks, while limb symptoms improved in 6-17 weeks.

**Conclusion:**

PDP should be considered in any child presenting with bulbar palsy/quadriparesis following previous history of fever/sore throat. Awareness and availability with timely administration of ADS within 48 hours are essential to reduce PDP, as antitoxin seems ineffective if administered after the second day of diphtheritic symptoms.

## Introduction

Post-Diphtheritic Paralysis (PDP), one of the most severe complications of diphtheria, is caused by exotoxin released by toxigenic strains of Corynebacterium diphtheria (1). Nontoxigenic strains commonly cause cutaneous diphtheria (1). PDP is reported to occur in 15% of faucial diphtheria infections in children (1). During 1998-2008, India accounted for 19%-84% of the global incidence of diphtheria. There were 5,125 diphtheria cases in 2000 and 4,233 in 2011 in India (1). Inadequate vaccine coverage in young children, waning vaccine-induced immunity in adults, mass population movements, poor living standards, delayed reporting to hospital, and non-availability or delay in administering antitoxin appear to be the main factors contributing to the re-emergence of the disease and high mortality (2). This study was planned to examine the current frequency of neurological complications of diphtheria.

## Materials & Methods

This was a retrospective observational study from January 2015 to December 2017. Ninety-one children identified as probable or confirmed diphtheria were evaluated, and among them, 35 children with post-diphtheritic palsy were identified. The patients’ demographic profiles, age, gender, and immunization status with calendar of events in their semiology were recorded. Children were considered completely immunized if they had received all the recommended doses of diphtheria toxoid, including booster, as per the national immunization schedule. All patients with a diagnosis of diphtheria were put on parenteral crystalline penicillin. Eleven out of 35 patients had received antitoxin between days 5-7 of illness. All the patients were managed in the isolation ward. Detailed clinical examination with appropriate investigations, including throat swab for staining and culture for Corynebacterium diptheriae, nerve conduction studies, electrocardiography, and echocardiography, was conducted, and results were tabulated and analyzed. Ethical clearance was obtained from the institutional ethical committee. 

## Results

A total of 91 children were identified as probable or confirmed diphtheria cases, and 35 were diagnosed with post diphtheritic paralysis. Among the 35 PDP cases, 23 (65.7%) were boys. Fifty-seven percent of patients with PDP were aged 6-10 years with a median age of presentation of 8.6 years, and they mostly (91.4%) belonged to rural areas of Karnataka and Andhra Pradesh. The immunization profile showed that of the patients, 13 (37.1%) were unimmunized, 12 (34.3%) were partially immunized, two (5.7%) were completely immunized, and eight (22.6%) had unknown status. The detailed demographic and clinical features of the patients are presented in Tables 1 and 2. 

All the 35 children had compatible clinical manifestations of diphtheria: fever with sore throat and dysphagia in the preceding 5-34 days prior to presentation. Nine (25.7%) patients had neck swelling with pseudomembrane. There were no cases of cutaneous diphtheria. The first PDP symptom was palatal palsy in all the 35 patients with a latency period of 10 (range 5-34) days after the onset of faucial diphtheria. Isolated bulbar palsy and palatal palsy followed by quadriparesis were seen in 20 (57.1%) and 15 (42.9%) patients, respectively. The median duration of progression of weakness was 10 days (range 7-15 days). Weakness was symmetric in 14 patients and asymmetric in one patient. Limb symptoms never preceded bulbar symptoms in any of the cases. Cranial nerves involved were nine and 10 in all the 35 patients, and the seventh nerve was additionally involved in one patient. Cerebellar ataxia was seen in five (14.3%) patients. 

Nine (25.7%) patients had evidence of co-existent myocarditis, while myocarditis with renal failure was seen in two (5.7%) patients. Three (8.6%) patients (of whom one child had respiratory muscle involvement) required mechanical ventilation for an average of three (range 1-6) days. Twenty-five (71.4%) patients required nasogastric tube nutrition. 

Eleven (31.4%) had received diphtheritic antitoxin between days 5-7 of illness. Of the 11 patients, eight developed quadriparesis (of whom five had myocarditis and another three developed cerebellar ataxia), and three developed isolated bulbar palsy. Of the remaining 24 patients who had not received diphtheritic antitoxin, 17 developed isolated bulbar palsy, and seven developed quadriparesis (with features of myocarditis in four patients).

Corynebacterium diphtheriae was seen in smear with throat swab culture positivity in five (14.3%) patients. Nerve conduction velocity (NCV) was performed in 11 children and revealed demyelinating neuropathy in seven children, axonal neuropathy in two children, and normal status in two children. CSF study was done on two patients, and results were unremarkable. Cardiac enzymes were grossly elevated in five patients, mildly elevated in two patients, and normally elevated in two patients. Four (11.4%) patients with PDP died, three from severe cardiomyopathy and one from aspiration, of whom three were unimmunized and one had unknown immunization status. All the four patients had received ADS between days 5-7 of illness.

Initial improvement of bulbar symptoms occurred by day 33 (range 15-50 days) after the onset of neurological symptoms. In the surviving 11 patients with quadriparesis, limb symptoms recovered by day 72 (40-120 days), with a muscle power grading of 3/5(MRC). At the time of discharge, 10 (28.6%) surviving patients with quadriparesis could not walk independently. The average duration of hospital stay was 25 (range 7-45) days.

## Discussion

There have been recent reports of sporadic diphtheria outbreaks from various states in India. In 2010, India shared 3123 (77.1%) of 4053 diphtheria cases reported to WHO (4), with an interesting trend of age shift from below 5 years to an older age group in recent studies (5,6). This has been attributed to decreased coverage with both DPT primary series and booster vaccination and decreased immunity in the community (7). In separate case series, Kanwal et al. (8) and Singh et al. (9) respectively reported 40% and 47.7% of their patients to be above 5 years of age while in a recent diphtheria outbreak in the state of Assam, most commonly affected individuals were aged more than 15 years (10). In the current study, 57% of patients were in the age group of 6-10 years, and males were the predominant gender. Similar findings of male preponderance were also seen in other studies (8, 11). This may be due to more concern about male children in our society. 

PDP is reported to be more common in unimmunized and partially immunized people, with limb involvement being more common and variable latency of disease in the unimmunized (10). Kanwal et al. (8) reported that in their study of 48 children, 15 were unimmunized, and 32 had incomplete immunization. Manikyamba D et al. (12) noted that 53% of their PDP cases were unimmunized, while 47% of them were partially immunized. In this study, we noted that 37.1% were unimmunized, 34.3% were partially immunized, 5.7% were completely immunized, and 22.9% had unknown immunization status. Also, quadriparesis and other diphtheria complications were observed to be more in the unimmunized (28.5%) than in the partially immunized (2.9%). The partially immunized (31.4%) presented more commonly with bulbar palsy without further progression to limb weakness compared to the unimmunized (8.6%).

The incidence of diphtheritic polyneuropathy is directly proportional to the severity of intoxication (1). PDP is more likely to occur in patients with severe infection, including those with “bull neck” and toxic shock. PDP occurs in about 10% of mild cases and 75% of severe cases (13). All the 35 patients in this study had fever with sore throat and dysphagia in the preceding 5-34 days prior to presentation, while nine (25.7%) patients had neck swelling with pseudomembrane comparable to the findings in two previous studies (13,14).

The latency period reported varies from 10 days to 3 months (15). The latency period noted in this study was 5-34 days. The clinical manifestations of myocarditis and neurological symptoms appear after a latent period of 10 to 14 days and between 2 to 7 weeks, respectively (16). The earliest manifestation of PDP is paralysis of the soft palate and posterior pharyngeal wall, which probably reflects the regional effects of locally produced toxin in faucal infection (17, 18, 19) and typically develops during the first 2 weeks in the form of palatal and pharyngeal paralysis (17). The peripheral neuropathy develops later, from 10 days to 3 months after the onset of oropharyngeal disease. Logina et al. (14) reported that in 28% of cases, limb symptoms started or continued to worsen while bulbar symptoms started to be improved. This biphasic fluctuation in the course of disease suggests that diphtheria toxin locally affects nerve endings in bulbar muscles at an early stage before systemic dispersion of the toxin produces late effects on the limb or bulbar nerves (14). The severity ranges from mild difficulty in walking to severe weakness and loss of ability to walk unaided (20). This biphasic course was similarly observed in 15 (42.9%) of our patients who developed weakness over 7-15 days following palatal palsy. 

 The involvement of other cranial nerves (II, V, XI, and XII) was described with varying frequency in various studies, ranging between 30%-84% of patients (14, 15) with more prevalence in adults (14, 15). In this study, all the patients had involvement of cranial nerves IX and X, and only one patient had facial nerve involvement. Kanwal et al. reported involvement of cranial nerves in 91.5% of cases, the nerves IX and X in 75% of the cases, the nerve III 4.1% of the cases, the nerve VI in 6.2% of the cases, and the nerve VII in 6.2% of the cases (8). Cerebellar ataxia may also be seen in some patients (15). Ataxia was seen in five (14.6%) of our patients.

There appears to be an inverse relationship between latency and recovery of motor symptoms – a longer latency corresponds with an earlier regression of limb weakness. In this study, limb weakness progression was seen in 42.9% of the cases while the rest 51.9% patients with bulbar palsy recovered without further progression to limb weakness. The reason behind isolated palatal palsy in children with no systemic involvement is probably lack of toxin dissemination (12).

Antitoxin administration aims to block cellular binding and uptake of diphtheria toxin. Although no formal clinical trials of antitoxin administration have been performed, the incidence of paralysis only seems to be reduced if antitoxin is administered during the first 2 or 3 days of diphtheritic infection (21). Antitoxin administered after day 2 of the infection does not modify the severity of neuropathy or prevent deaths (14, 16). This highlights the importance of immediate administration of ADS at the earliest point of diagnosis.

Segmental demyelination of peripheral nerves is the characteristic lesion, with additional axonal degeneration in the most severe cases (22). Electrophysiological studies show a maximum impairment at weeks 3–10 and then gradual improvement. Abnormal studies may persist beyond 100 days after the onset of polyneuropathy and occasionally even up to a year later (20). In this study, demyelinating neuropathy was noted in 60% of the children. 

Although throat cultures provide an accurate means for diagnosis of diphtheria (1), isolation of the bacterium from a throat swab specimen is unlikely in most cases (2). In addition, false-negative results may be obtained if there is a delay in processing the specimen (16). Corynebacterium diphtheriae was seen in smear with throat swab culture positivity in five (14.3%) patients in this study, while Kanwal et al., in their study reported culture positivity in two (4.2%) patients. Other reports of diphtheritic neuropathy in India have been bacteriologically confirmed for 15%–39% of patients (9, 23, 24). Indiscriminate use of antimicrobial drugs before the diagnosis of diphtheria may make culture positivity even more complex (23). 

Cerebrospinal fluid (CSF) studies may be normal or show elevated protein (albumin-cytologic dissociation). Logina et al. reported elevated CSF proteins in 12% of their PDP patients (14). CSF study performed on two of our patients had normal results.

We noted initial improvement of bulbar symptoms by day 33 (range 15-50 days) after the onset of neurological symptoms and improvement in limb weakness to 3/5 by day 72 (40-120 days). At the time of discharge, 10 (28.6%) surviving patients with quadriparesis could not walk independently. Mortality was 11.43% in this study, causing cardiogenic shock secondary to post-diphtheritic myocarditis, while Kanwal et al. (8) reported mortality as 14.6% in their study. Similar to Kanwal et al. (8), we also found no significant difference in the progression of the disease, the complication rate, or recovery between children less than and greater than 5 years of age. The limitation of this study was its retrospective nature. The strength lies in the fact that this is one of the largest data on PDP in the pediatric population from Bangalore, Karnataka.

**Table 1:** Immunization status and clinical profile of study population

**Immunization status (N=35)**	**Bulbar palsy (N=20)**	**Quadriparesis (N=15)**	**Myocarditis (N=9)**	**Mechanical ventilation (N=3)**	**Survived ** **(N=26)**	**Death ** **(N=4)**
**Completely immunized (N=2 )**	2	-	-	-	2	-
**Partially immunized (N=12 )**	11	1	1	1	11	-
**Unimmunized (N= 13)**	3	10	6	2	10	3
**Unknown(N=8 )**	4	4	2	-	3	1
**Total(N= 35)**	20	15	9	3	26	4

**Table 2:** Comparison of demographic and clinical profile of study population with previously reported

**Total no. of patients**	**Present study** **(n=35)**	**Kanwal et al** **(n=48)**	**Manikyamba et al(n=13)**	**AFP surveillance** **(n=15)**	**Logina et al** **(n-50)**
**Gender:** Male: Female	23:12	36:12	4:9	9:6	26:24
**Age group (years**)	3.9-17	1.5-11	5-13	<15	41-60
**Immunization status**					
Completely immunized	2(5.7%)	-	-	-	-
Partially immunized	12(34.3%)	33(68.7%)	47%	-	-
Unimmunized	13(37.1%)	15(31.3%)	53%		
Unknown	8(22.6%)	-	-	-	
**Membranous tonsillitis**	9	48			30(60%)
**Anti-toxin**	11	-	-	-	33
**Latency period**	5-34days	5–45 days	15-30days	-	10
**Isolated Palatal palsy **	20(57.1%)	25(52%)	60%	15%	10%
**Limb weakness**	15(42.9%)	23(48%)	40%	85%	90%
**Cranial nerve involvement **	35(100%)	44(91.6%)	-	-	-
**Respiratory muscle involvement **	1(2.6%)	41 (85.4%)	-	-	-
**Requiring mechanical ventilation **	3(8.6%)	29 (60.4%)	none	20%	10(20%)
**Duration of ventilation( days)**	3(1-6)	13	-	-	27(3-64)
**Myocarditis**	9(25.7%)	20 (41.7%)	-	-	32(64%)
**Nerve Conduction Study **	Done in 11 Demyelination Motor neuropathy in 7 and axonal 2 and normal in 1	Done in 12 Demyelination Motor neuropathy in 4 and axonal 3 and combined in 5	Done in 5 Demyelinating in 2 and axonal degeneration in 4	-	Distal motor latencies were prolonged
**C.diphtheriae isolated**	5(14.3%)	2(4.2%)	-	-	38
**Recovery time (days)**					
Gag reflex recovery time ( median range in days)	35 (21-50 )	16 (7–50)	2-4weeks	-	3-98
Limb recovery time (till power -3/5) ( median range in days)	72( 40-120)	18 (8–48)	5-6weeks	-	73(20-115)
**Outcome**					
Death	4(11.4%)	14.6%	8%	46%	8(16%)

## In Conclusion

PDP should be considered in the differential diagnosis of acute flaccid paralysis, especially in children presenting with bulbar palsy/quadriparesis following previous history of fever and sore throat. Timely diagnosis and differentiation from other neuropathies are important for rational management. Awareness of regular immunization, both primary and booster DPT vaccination, is the need of the hour as it is the simplest, most cost-effective practical approach in preventing complications due to this vaccine-preventable disease. 

## Author’s Contribution

VK: Concept and designed the study

SV: Collected the data and helped in data analysis. 

SK: Analyzed data and drafted the manuscript

NB: Literature search, Concept of study and supervised seizure control

AB: Edited and refined draft

## Conflict of interest

None of declare.
